# The ESKAPE Challenge: Understanding Resistance and Exploring Alternative Treatments

**DOI:** 10.3390/antibiotics15060550

**Published:** 2026-05-29

**Authors:** Kartika Vashishtha, Pobitra Borah, Robert Sonowal

**Affiliations:** 1 Department of Development Biology and Genetics, Indian Institute of Science, C.V. Raman Avenue, Bangalore 560012, Karnataka, India; 2 Department of Biological Sciences and Bioengineering, Indian Institute of Technology Kanpur, Kanpur 208016, Uttar Pradesh, India; pborah22@iitk.ac.in; 3 Department of Pharmacology, Pratiksha Institute of Pharmaceutical Sciences, Chandrapur Road, Panikhaiti, Guwahati 781026, Assam, India

**Keywords:** antimicrobial resistance (AMR), ESKAPE pathogens, fecal microbiota transplantation (FMT), methicillin-resistant *Staphylococcus aureus* (MRSA), vancomycin-intermediate *Staphylococcus aureus* (VISA), vancomycin-resistant *Enterococci* (VRE), vancomycin-resistant *Staphylococcus aureus* (VRSA)

## Abstract

Antimicrobial resistance (AMR) constitutes a critical and escalating global public health challenge, severely limiting the potential of existing antimicrobial drugs and escalating infection-associated morbidity and mortality rates. This analysis focuses on the ESKAPE pathogens (*Enterococcus faecium*, *Staphylococcus aureus*, *Klebsiella pneumoniae*, *Acinetobacter baumannii*, *Pseudomonas aeruginosa*, and *Enterobacter* species), which are prioritized by the World Health Organization (WHO) and represent a significant cause of nosocomial infections due to their extensive drug resistance. We provide an in-depth review of the global prevalence and specific antibiotic-resistant mechanisms of these pathogens. Due to the decline in the traditional antibiotic development pipeline, accelerated development of alternative therapeutic strategies is essential. The review comprehensively discusses innovative non-traditional therapies currently being explored to bypass traditional antibiotic limitations, such as phage therapy, antimicrobial peptides (AMPs), anti-virulence therapies, fecal microbiota transplantation (FMT), and targeted CRISPR-based approaches. Addressing the ESKAPE challenge requires a concerted, multi-sectoral strategy guided by the One Health principle, focusing on enhancing public awareness, improving surveillance and research, optimizing judicious antibiotic use, and cultivating sustainable investment in novel interventions.

## 1. Introduction

Introduction of antimicrobial drugs in the mid-twentieth century revolutionized the health-care sector by significantly reducing the risk of infections associated with injuries, childbirth, and intrusive surgical procedures [1]. However, euphoria surrounding the rise in antimicrobials gradually diminished over time due to the alarming emergence of antimicrobial resistance (AMR) in clinical settings. The escalation of drug resistance acquired by the pathogens can be ascribed to injudicious antimicrobial use, inappropriate drug or dose selection, poor treatment adherence, and lack of effective diagnostic methods [2,3]. While mismanagement accelerates the crisis, the global rise in AMR is also an inherent consequence of the widespread adoption of antibiotic therapies and expanded access to healthcare. This successful integration of antimicrobials into modern medicine provides a persistent, large-scale selective pressure on microbial populations, making resistance a global challenge even alongside appropriate clinical use. The advent of AMR is limiting the potential of existing drugs and other treatment options, thereby increasing the risk of illness spread and infection-associated death. In addition to morbidity and mortality, AMR also possesses a significant economic burden attributable to prolonged hospital stays, expensive medicines, and complex drug regimens [4]. Misuse and overuse of existing antibiotics have resulted in the accelerated development of bacterial resistance, surpassing the pace at which novel drugs can be developed. Consequently, the emergence of AMR has been accompanied by a decline in the antimicrobial development pipeline and a shortage of innovative drugs. In response to this AMR challenge, the World Health Organization (WHO) published a list of antibiotic-resistant “priority pathogens” in 2017 and 2024 in order to encourage intensive research and development against antimicrobial-resistant organisms [5,6,7]. The pathogens collectively known as ESKAPE (*Enterococcus faecium*, *Staphylococcus aureus*, *Klebsiella pneumoniae*, *Acinetobacter baumannii*, *Pseudomonas aeruginosa*, and *Enterobacter* species) were mentioned in the WHO bacterial priority pathogens list [7]. In this list, antibiotic-resistant ESKAPE pathogens, mainly Gram-negative bacteria carbapenem-resistant *A. baumannii* (CRAB) and *P. aeruginosa* (CRPA), and third-generation cephalosporin-resistant *K. pneumoniae* (CRKP) and *Enterobacteriaceae* (CRE), and Gram-positive bacteria, including vancomycin-resistant *E. faecium* (VRE) and methicillin-resistant (MRSA) and/or vancomycin intermediate/resistant *S. aureus* (VISA/VRSA), were ranked as the most important pathogens [7,8,9].

ESKAPE pathogens account for substantial numbers of nosocomial infections, and they make up the vast majority of isolates whose antimicrobial resistance poses significant therapeutic challenges in clinical practice [10,11]. Furthermore, a recently published study showed that ESKAPE pathogens developed resistance within 60 days of an evolutionary experiment conducted in vitro [12]. While antibiotics are crucial for fighting infections, their widespread use has led to significant challenges, such as growing resistance, collateral damage to the microbiome, and the development gap. The process of discovering, developing, and getting a new antibiotic to market is long and expensive. Consequently, antibiotic research and development has seen a significant decline due to low return on investment for pharmaceutical companies, creating a critical gap in the exploration of therapeutic options [13,14]. Therefore, accelerated development of alternative strategies is essential to address the issue. They focus on different approaches that can bypass the limitations of traditional antibiotics, such as circumventing resistance mechanisms, targeting specific pathogens, enhancing host immunity, and using a combination of antibiotics. Through this survey, we aim to provide in-depth knowledge about ESKAPE pathogens, their antibiotic-resistant patterns, and newly discovered traditional and alternative therapies to fight them. In this analysis, we have thoroughly discussed the prevalence and antibiotic-resistant mechanisms of Gram-positive (VRE, MRSA, VISA/VRSA) and Gram-negative (CRAB, CRE, CRKP, and CRPA) antibiotic-resistant ESKAPE pathogens, along with the scientific and socioeconomic measures that need to be adopted to combat them.

## 2. Prevalence and Mechanisms of Antibiotic Resistance in ESKAPE Pathogens

**Figure 1:**
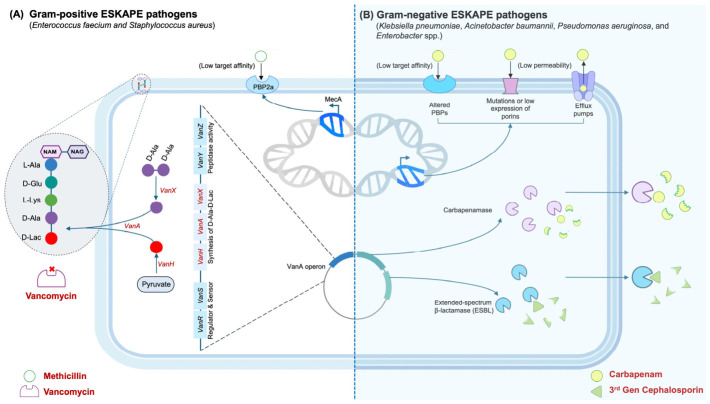
Resistance mechanisms of antibiotic-resistant ESKAPE pathogens. (**A**) In Gram-positive pathogens (*E. faecium* and *S. aureus*), vancomycin resistance is mediated by the *vanA* operon by altering the peptidoglycan synthesis via replacing the terminal D-Ala–D-Ala with D-Ala–D-Lac to cause a marked reduction in drug binding affinity. Whereas methicillin resistance in *S. aureus* is primarily conferred by the *mecA* gene that encodes for the low-affinity PBP2a, which results in cell wall synthesis even in the presence of β-lactam antibiotics. (**B**) In the case of Gram-negative pathogens (*K. pneumoniae*, *A. baumannii*, *P. aeruginosa*, and *Enterobacter* species), drug resistance is mostly mediated via the following mechanisms: (i) production of β-lactamases, such as ESBLs capable of hydrolyzing third-generation cephalosporins and carbapenemases capable of inactivating carbapenems; (ii) expression of low-affinity PBPs; (iii) decreased membrane permeability due to porin loss or modification; and (iv) expression of efflux pumps.

Infections associated with antibiotic-resistant ESKAPE pathogens (VRE, MRSA, VISA/VRSA, CRAB, CRE, CRKP, and CRPA) are a leading global cause of mortality and morbidity. The clinical isolates of these pathogens are linked to several potentially fatal hospital-acquired infections (HAIs), like pneumonia, meningitis, bacteremia, wound infections, and urinary tract infections (UTIs) in ICUs [15]. The molecular mechanisms of antibiotic resistance adopted by these pathogens are shown in Figure 1.

## 3. Vancomycin-Resistant Enterococci (VRE)

Prevalence: Enterococci are Gram-positive chains/pairs of cocci, facultative anaerobes. Their abundance in the gastrointestinal (GI) tract of humans, animals, and insects, fermented products, water, plants, and soil, contributes to their remarkable resilient capacity of enduring harsh conditions [16]. The two most clinically relevant species of human intestines are *E. faecalis* and *E. faecium,* as they cause the majority of HAIs [17]. Both strains show varying degrees of vancomycin resistance. Importantly, vancomycin and its analogs are regarded as the last resort treatment for severe infections caused by Gram-Positive bacteria, including Enterococcus species [18]. Consequently, the dissemination of resistance mechanisms within pathogenic species is another threat to the clinical efficacy of vancomycin [19]. Many VRE infections cause serious and often fatal disease [20,21,22]. In a hospital setting, *Enterococci* have been referred to as “triple-threat” pathogens because they can colonize with Enterobacteriaceae and *S. aureus* in the gut and skin, respectively [23,24]. According to the German Antimicrobial Resistance Surveillance System, vancomycin-resistant *E. faecium* increased from 16.2% in 2008 to 18.5% in 2012 under hospital settings [25]. Similarly, the European Antimicrobial Resistance Surveillance Network (EARS-Net) reported that vancomycin-resistant *E. faecalis* invasive isolates increased from 10.4% to 17.3% within four years (2014–2018) [26,27] have reported 8.10% of VRE prevalence in Asia. Subgroup analysis revealed that VRE are highly prevalent in Western Asia (11.4%), followed by South Asia (7.70%), East Asia (3.10%), and Southeast Asia (1.80%) [27]. Overall, the prevalence of VRE (both in humans and animals) rose from 0.05% to 99.0% globally, and in India, it grew alarmingly from 1.0% to 45.6% in the last two decades [28]. A very recently published work predicted using a Bayesian age-period-cohort model that vancomycin-resistant *E. faecium* burden will continue to rise by 2050, while vancomycin-resistant *E. faecalis* will sustain a decline [29].

Mechanisms of resistance against vancomycin: Vancomycin is a tricyclic glycopeptide cell wall synthesis inhibitor, which disrupts the peptidoglycan assembly by inhibiting the late-stage peptidoglycan synthesis. Mechanistically, it forms hydrogen bonds with the second-to-last D-Ala4-D-Ala5 dipeptide of newly synthesized UDP-N-acetylmuramyl-pentapeptides, and molecularly, it prevents trans-glycosylation and transpeptidation processes catalyzed by penicillin-binding protein 2a (PBP2a) and PBP2a enzymes, respectively [30]. Subsequently, the accumulation of bound vancomycin-pentapeptide complexes within the cell stops the Gram-positive cell from surviving. In the late 1980s, the heavy prescription of vancomycin to treat or prevent Gram-positive infections (MRSA) led to a rise in VRE in US hospitals [31]. The resistance was conferred by vancomycin-dependent activation of either *vanA* or *vanB*, two functionally similar operons (the *vanA*-mediated resistance mechanism is shown in Figure 1) [32,33]. The *vanA* operon encodes seven genes, namely *vanH*, *VanA*, *vanX*, *vanS*, *vanY*, *vanR*, and *vanZ*, and is regulated by *vanS* and *vanR* (two-component regulatory system). The *vanA* (a ligase) and *vanH* (a dehydrogenase) are involved in the synthesis of depsipeptide D-Ala-D-Lac. While *vanA* mediates esterification of the D-Ala-D-Lac depsipeptide, *vanH* produces D-Lac by pyruvate reduction [34]. Further, *vanX* (a D,D-dipeptidase) hydrolyzes the ester bond of D-Ala-D-Ala and ensures that the newly formed depsipeptide does not compete to bind to UDP-linked tripeptide peptidoglycan precursor [35]. Lastly, *vanY* (D, D-carboxylpeptidase) facilitates the cleavage of D-Ala-D-Ala dipeptides attached to the C-terminal of pentapeptides. The role of *vanY* is non-essential for conferring resistance [36]. However, *vanZ* is involved in conferring resistance to teicoplanin (vancomycin homolog) [37,38]. Evolutionary studies showed that resistant genes were acquired by enterococci from other species [39,40]. Researchers speculate that oral administration of vancomycin led to such high resistance in the community, as these agents are not well absorbed in the intestine, resulting in higher fecal vancomycin concentrations, which often are not enough to kill the tolerant enterococcus and also favor the colonization of naturally glycopeptide-resistant species such as *streptomycetes* (fungi) in the gastrointestinal tract. The damage to the microbiota caused by extensive use of vancomycin leads to the domination of VRE in the intestine. In fact, many commonly used antibiotics, such as aztreonam, aminoglycosides, cephalosporins, clindamycin, penicillin, nafcillin, oxacillin, and trimethoprim-sulfamethoxazole, have contributed towards the increase in the appearance of VRE strains [39,41,42,43].

## 4. Methicillin-Resistant *S. aureus* (MRSA) and Vancomycin-Intermediate/Resistant *S. aureus* (VISA/VRSA)

Prevalence: *S. aureus* is a Gram-positive, round-shaped (coccus) bacterium, frequently found as a part of the human skin microbiota. They can survive on both aerobic and anaerobic respiration. Once *S. aureus* strains enter the human body, they can lead to severe infections, like pneumonia, osteomyelitis, endocarditis, arthritis, sepsis, and toxic shock syndrome [24]. Moreover, its ability to form biofilms further adds to the complication while giving antibiotic therapy to the patients. In the current settings, *S. aureus* has become resistant to several commonly used antibiotics. Within two years of the introduction of methicillin (1959), for the treatment of penicillin-resistant *S. aureus*, the first case of MRSA was reported in England [44]. The widespread presence of MRSA in the 1980s led to the accelerated usage of vancomycin in health care as empiric treatment for staphylococcal infections. During this time, the growing cases of *Clostridium difficile* and coagulase-negative staphylococci infections in the U.S. also led to increased consumption of vancomycin [45,46]. In the 1990s, a strong selective pressure was established, which led to the emergence of strains (VISA/VRSA) less susceptible to glycopeptides, including vancomycin [47]. The first strain of vancomycin-resistant *S. aureus* (VRSA) was isolated in the year 2002 in the U.S. [48], and strains of vancomycin-intermediate *S. aureus* (VISA) have been reported from across the globe [47,49,50,51]. To understand the statistical prevalence of vancomycin in *S. aureus,* an in-depth meta-analysis was performed, demonstrating that before 2010, the overall prevalence of VRSA and VISA was 1.2%, and it had reached 2.4% and 4.3% in 2020. The frequency of VRSA and VISA has increased 2- and 2.6-fold, respectively, over the years as compared to what it was prior to 2010. Results from subgroup analysis show that VISA is highly prevalent in Asia as compared to the United States, with a frequency of 2.1%. However, VRSA occurs with the highest frequency in the USA, which is approximately 3.6% [52]. Hasanpour et al. have shown that the global prevalence of MRSA was 14.69% in 2023. In this analysis, 119 studies were considered (164,717 participants from 29 different countries) [53]. Antibiotic-resistant *S. aureus* ranks second among other leading pathogens for antimicrobial resistance-associated deaths [54].

Resistance Mechanisms: MRSA—Methicillin, a β-lactam antibiotic, inhibits the binding of penicillin to penicillin-binding protein (PBP), which is essential for the peptidoglycan layer synthesis in the bacterial cell wall [55]. MRSA is caused by two genes, *mecA* and *mecC,* which code for PBP2a and PBP2c enzymes. Both genes confer resistance against β-lactam antibiotics, like penicillin G (penicillinase-labile), methicillin (penicillinase-stable), and cefoxitin (a cephalosporin) [56]. It was further validated that *mecC*-MRSA was naturally selected in the wild (pre-antibiotic era) and not due to the clinical abuse of antibiotics, as β-lactam antibiotics producing dermatophyte *Trichophyton erinacei* provided the necessary selective pressure in the natural environment, in which MRSA was at a growth advantage over its susceptible counterpart [56]. Later, the extensive usage of antibiotics in livestock imparted a selective advantage for MRSA to emerge in domesticated animals, which later jumped to humans. This is supported by the discovery and isolation of the *mecC*-MRSA gene from domesticated cows in Europe initially. Also, studies from different parts of Europe confirmed that a low frequency of MRSA was found in other domesticated animals such as goats, horses, and sheep, etc. [57,58,59].

Resistance Mechanisms: VISA and VRSA—To date, at least eleven van gene clusters have been identified to confer vancomycin resistance, *viz*. *vanA*, *vanB*, *vanD*, *vanF*, *vanI*, *vanM*, *vanC*, *vanE*, *vanG*, *vanL*, and *vanN* [60]. Of the eleven genes, only the *vanA* cluster has been identified to be associated with resistance in isolated VISA/VRSA strains. VISA shows intermediate resistance (MIC = 4–8 µg/mL), while VRSA exhibits full resistance to the drug (MIC > 16 µg/mL), making them difficult to treat with standard antibiotic regimens [61]. The strain acquired the *vanA* operon encoded on transposon *Tn1546*, which was originally associated with the VRE conjugative plasmid, and *S. aureus* must have acquired enterococcal plasmids during conjugation [62,63,64]. The vast majority of clinical VRSA isolates harbor the *vanA* operon. The presence of the resistant gene *vanA* in *S. aureus* represents a serious epidemiological concern, given its ability to acquire and express resistance genes from the *Enterococcus* reservoir.

## 5. Carbapenem-Resistant *Acinetobacter baumannii* (CRAB)

Prevalence: *A. baumannii* is a Gram-negative, non-motile, aerobic coccobacillus commonly found in natural environments like soil. It can also colonize the skin, lungs, urinary tract, and bloodstream and cause infections in individuals with a weak immune system. Reports showed that most of the HAIs, such as skin, UTIs, soft tissues, and bloodstream infections, are caused by *A. baumannii*. Therefore, it emerged as an opportunistic pathogen of global importance in humans due to its high incidence among patients experiencing prolonged hospital stays (>90 days) [65]. The remarkable ability to survive harsh conditions and develop high resistance makes it extremely difficult to treat in clinical settings. It can grow on all surface types, in the presence of ethanol as a sole carbon source, tolerate oxidative stress by enhancing the catalase expression, and also produce biofilms, which makes it even more virulent [66]. Over the period of time, *A. baumannii* has developed resistance against different antibiotic classes, including carbapenems, which are often used as a last resort option [67]. In general, carbapenems, e.g., imipenem, panipenem, doripenem, meropenem, ertapenem, and biapenem, are β-lactam antibiotics that exhibit efficacy against extended-spectrum β-lactamase (ESBL)-producing bacteria [68]. CRAB infections are considered a public health concern given their resistance to these last-resort antibiotics. Historically, only three European clones of *A. baumannii* (EUI, EUII, and EUIII) were found to cause outbreaks in the 1980s and early 2000s, particularly in Europe [69,70]. Lately, most of the CRAB infections worldwide have been attributed to just a few widespread *A. baumannii* lineages. Only recently, in 2023, Müller et al. published the data wherein they have sequenced 313 CRAB isolates from 114 hospitals across the globe, including Africa, Asia, Europe, and North and Latin America (47 countries). They discovered that 92.3% of CRAB isolates were assigned to the commonly present IC1 (international clone 1) to IC8 (international clone 8). Among the list, IC2 dominated the world with approximately 62.6%, followed by 14.1% clone 5 (IC5), which was limited to Latin America only. Approximately 1.9% of the total isolates representing the novel international clone 9 (IC9) were found to originate in Italy, Belgium, Egypt, and Pakistan [71]. A very recent report shows that sputum-derived CRAB isolates of pneumonia patients from southern Thailand are dominated by the high-risk IC2 lineage and *bla_OXA-23_*-driven resistance [72].

Resistance Mechanisms: Carbapenems are broad-spectrum β-lactam antibiotics used against both Gram-negative and Gram-positive bacteria. They are either semi-synthetic or structurally like penicillin. When other antibiotics fail to cure the infections, carbapenems are used as the last line of defense [73]. A few examples of carbapenems are imipenem, meropenem, ertapenem, tebipenem, doripenem, panipenem/betamipron, and biapenem. Heavy misuse of carbapenems led to the rise in antimicrobial resistance in the clinical setting. Modifications in penicillin-binding proteins (PBPs), reduced permeability due to functional loss of porins in the outer membrane (mutations or low expression), overexpression of efflux pumps, production of carbapenemase or β-lactamases (AmpC and ESBLs), and acquired resistance from genetic elements like transposons, conjugative plasmids, some of the ways through which *A. baumannii* confers resistance against carbapenems [74,75]. The three major resistance mechanisms are described as follows: (a) by alteration in PBPs, (b) reduction in permeability, and (c) by increasing production of carbapenemase and β-lactamases. Studies have shown that carbapenem resistance due to alterations in PBPs is only modest [76] and does not contribute to CRAB infections significantly. Modification in porins and outer membrane proteins (OMPs) also alters the transportation of antimicrobials inside the cells. In *A. baumannii*, CarO (carbapenem-associated OMP), OmpW, and HMP-AB are found to be associated with trafficking of β-lactams across the membrane, resulting in reduced meropenem and imipenem susceptibility. Many CRAB clinical isolates contain mutations, which either cause structural defects or complete loss of the CarO protein, indicating its concrete role in carbapenem resistance [77,78]. Finally, high production of carbapenemases and β-lactamases inactivates or degrades carbapenems. The carbapenemases belonging to the OXA type (*bla_OXA-23_* and *bla_OXA-24/40_*) are commonly present on transmissible plasmids. Both enzymes are responsible for causing high mortality rates. Four metallo-β-lactamases are commonly found and identified in *A. baumannii*, namely, IMP (imipenemase), SIM (Seoul imipenemase), VIM (Verona integron-encoded metallo-β-lactamase), and NDM (New-Delhi metallo-β-lactamase). Out of these, VIM and IMP confer the majority of resistance against β-lactams [79,80].

## 6. Carbapenem-Resistant *Pseudomonas aeruginosa* (CRPA)

Prevalence: *P. aeruginosa* is a rod-shaped, non-spore-forming Gram-negative bacterium commonly found in freshwater bodies such as swimming pools in urban settings and in cleaning solutions, sinks, icemakers, soaps, and endoscopes, etc., in medical settings. Like other opportunistic pathogens, *P. aeruginosa* also infects hosts with compromised immune systems, such as individuals suffering from cystic fibrosis, AIDS, respiratory issues, burns, organ transplants, and ICU patients staying for longer durations. Since the organism is widely present in aquatic environments, it can lead to several community-acquired infections like eye and ear infections, osteomyelitis, pneumonia, etc. [81]. It has evolved a variety of mechanisms to resist antibiotics in its surroundings, such as rapid mutations, alteration of membrane permeability to restrict the entry of harmful substances (including antibiotics), and production of β-lactamases to cleave β-lactams. *P. aeruginosa* in biofilm states can survive in other extremely harsh environments, which increases its potential to resist host defense mechanisms and tackle antibiotics [81]. Over the last 20 years, *P. aeruginosa* infections, including bacteremia and pneumonia, have increased exponentially with high morbidity and mortality rates. Approximately ten *P. aeruginosa* lineages (ST111, ST175, ST233, ST235, ST244, ST277, ST298 (CC445), ST308, ST357, and ST654) that showed global dominance and multi-drug resistance phenotype have been identified using sequence typing techniques [82]. This dual threat of widespread presence and resistance is what makes these clones particularly dangerous. Among these, ST235 and ST111 are particularly concerning because they produce a wide range of carbapenemases, including classes A, B, and D. According to CHINET (China Antimicrobial Surveillance Network), *P. aeruginosa* is the most commonly isolated bacillus (7.96%) of healthcare-associated infections [83]. A retrospective case-controlled study determined 528 *P. aeruginosa* cases in ICU patients over six years (from January 2016 to December 2021), in which CRPA was found in 18.4% of cases, and multidrug-resistant *P. aeruginosa* (MDRPA) was found in 25.6% of cases. Longer hospitalization (more than 28 days), invasive surgeries, and blood transfusions within 30 days before infections were some of the major factors for causing CRPA infections. This study positively pointed out that having a birth weight of more than 2500 g and breastfeeding can combat CRPA infections [84]. In Taiwan, CRPA infections have increased from 12% (2012–2015) to 19–23% (2018–2021) [85]. Similarly, the Indian Council of Medical Research (ICMR) also reported increasing prevalence of CRPA (42–47%) in Indian hospitals. A regional-level study conducted in northern India showed a significantly higher CRPA prevalence of 72.5% among hospitalized patients [86]. In the USA and the EU, the MDR strains of *P. aeruginosa* prevalence was approximately 15% and 17%, respectively [87,88]. Among all the antibiotic-resistant strains of *P. aeruginosa*, carbapenemase-producing CRPA (CP-CRPA) poses a major threat to society as individuals infected with CRPA are difficult to treat. In 2021, the Antimicrobial Resistance Laboratory Network revealed 280 cases of CP-CRPA, the majority of these were having VIM type β-lactamase. However, in Eurasia and the Middle East, the most prevalent type is NDM-CRPA [89]. Overall, the epidemiological studies showed that *P. aeruginosa* poses a global threat to human health.

Resistance Mechanisms: The introduction of imipenem and meropenem in the health care sector led to the emergence of MDRPA and CRPA strains. Imipenem resistance is primarily due to inactivation of the bacterial outer membrane protein. While meropenem resistance is due to higher expression of efflux systems, which pump the drug out [90]. However, CRPA is far more complicated and stems from resistance by combining different mechanisms, including porin mutations, increased MexA-MexB-OprM efflux pump activity, elevated production of β-lactamases (particularly AmpC), alterations to other bacterial PBPs, and inactivation of OprD [91]. The gene *bla*_VIM_ is present on mobile genetic elements, such as incompatibility-type plasmids and class 1 integrons. These elements allow the resistance to spread easily between bacteria and among other species. The primary driver of the increase in CRPA emergence is due to the MBL production, as it breaks down carbapenem antibiotics. Out of the lot, VIM-MBLs appeared as a major culprit. The most isolated MBL genes, *bla*_VIM-2_ and *bla*_VIM-4_, are located within class 1 integrons that further facilitate their spread. In addition to VIM MBLs, other carbapenemases also contribute to CRPA infections, such as Ambler Class A, which includes *K. pneumoniae* carbapenemase (KPC) and Guiana extended-spectrum (GES), and Ambler Class B (MBLs), which includes VIM, IMP (imipenemase), and NDM-MBL. The NDM-type carbapenemase is extremely dangerous because it has a very wide range of activity and can break down almost all beta-lactam antibiotics [92,93]. This bacterial resistance not only makes carbapenems ineffective but also reduces the effectiveness of other antibiotics like aminoglycosides and fluoroquinolones. Furthermore, it diminishes the effectiveness of other antipseudomonal drugs, including the older ones like cefepime, ceftazidime, and piperacillin-tazobactam, and even newer β-lactam/β-lactamase inhibitors (BL-BLI) combinations, like imipenem-relebactam, ceftolozane-tazobactam, and ceftazidime-avibactam [94].

## 7. Carbapenem-Resistant Enterobacterales (CRE)

Prevalence: Enterobacterales is the largest order of bacteria that includes many common gut bacteria. Some of the species belonging to this order are considered major threats to the community, as they readily cause infections in suitable environments. Few members of Enterobacterales are also part of the ESKAPE pathogens list, namely, CRKP, *Klebsiella aerogenes* (previously termed *Enterobacter aerogenes*), and *Enterobacter cloacae* (CREC). Although *E. coli* is not a member of ESKAPE, it is now recognized as a significant threat [95]. These bacteria commonly cause UTIs, pneumonia, bacteremia, and other intra-abdominal infections. The CDC estimated that in 2017, CRE were responsible for 13,100 infections and 1100 deaths among hospitalized patients in the U.S. While comprehensive research is still needed to consolidate the total global and Indian morbidity and mortality data for all antibiotic-resistant Enterobacterales, studies in India highlight the severity of the issue. For instance, CRE colonization in one Indian ICU was specifically associated with a 27% mortality rate [96]. Importantly, *K. pneumoniae* infections exhibiting carbapenem resistance have become increasingly common, surpassing 50% in some areas of Europe and the Eastern Mediterranean [97,98]. After its initial report in the US in the early 2000s, CRKP has become a worldwide health concern. This resistance has gradually spread across the globe, including the USA, South and Latin America, Southeast Asia, China, the Mediterranean, Africa, and various European regions. CRKP-dominated areas have reported 20–40% of HAIs [99]. The highest rates of carbapenem resistance are particularly found in Russia, Greece, China, India, Saudi Arabia, Peru, Argentina, Iran, Italy, and Brazil [100]. A recent meta-analysis indicates that global CRKP colonization frequency varies widely (0.13–22%), averaging 5.43%, while incidence ranges even more broadly (2–73%), averaging 22.3% [101]. In Europe, 687 CRKP strains from 41 hospitals across nine Southern European countries (between 2016 and 2018) were examined, and 11 major lineages, predominantly ST258/512, ST101, ST11, and ST307, were identified [102]. The most common resistance genes were *bla*_KPC_-like (46%) and *bla*_OXA-48_ (39%), with the study revealing regional differences in the spread of high-risk clones and specific carbapenemase genes across Europe when compared to an earlier collection. CDC reported a greater than 35% surge in hospital-onset CRKP infections in the US from 2019 to 2020 [103]. The problem is escalating in low/middle-income countries, given their vulnerable conditions and limited resources [104]. The Enterobacter cloacae complex (ECC) is mostly recognized as a potential cause of HAIs. ECC organisms fall into two main groups: an older, more diverse clade and a newer, more closely related clade linked to healthcare settings [105]. A UK study of bloodstream isolates from 2001–2011 suggested these two groups diverged 50–250 years ago. Their widespread distribution, rather than limited geographic clustering, implies they were present in community microbiota before becoming hospital-acquired [106]. Unlike CRKP, CREC consists of a small group of high-risk isolates, including ECC III (ST78), *E. hormaechei* subsp. *steigerwaltii* (ST90/93) and *E. xiangfengensis* (ST171/114). Multiple sequence types in each region share the same mobile genetic elements and carbapenem resistance genes. The distribution of these resistance enzymes varies geographically: VIM is highly prevalent in Europe, NDM in Southeast Asia and South America, OXA-48-like in North Africa and the Middle East, KPC in the Americas, and IMP in the Pacific [107]. The dominant ST171 strain first appeared in the Northeastern United States. It acquired either the *bla_KPC-3/4_* gene on IncHI2/IncHI2A or IncFIA plasmids, respectively, before spreading clonally throughout the American Midwest and Mid-Atlantic regions [108,109]. The diverse and varied resistance mechanisms of CREC make it especially challenging for hospitals to prevent infections. The natural presence of members of Enterobacterales in the gut, which can acquire and spread resistance genes and cause common infections, collectively designates them as a significant public health concern.

Resistance Mechanisms: The primary mechanism driving resistance in Enterobacterales involves β-lactamase production, specifically carbapenemases and ESBLs. ESBLs, such as the CTX-M types, can break down common β-lactams like penicillins and cephalosporins. More critically, carbapenemases—including serine carbapenemases like OXA-48 and KPC and MBLs like VIM, NDM, and IMP, etc.—degrade carbapenems, which are often used as last-line antibiotics [110]. *E. coli,* being naturally susceptible to several antimicrobial compounds, is emerging as a concerning pathogen considering its ability to transfer resistance horizontally through plasmids, integrons, and transposons. The key resistance mechanisms in *E. coli* are production of ESBLs, carbapenemases, 16S rRNA methylases (that confer aminoglycoside resistance), plasmid-mediated quinolone resistance (PMQR) genes, and *mcr* genes (that confer polymyxin resistance, including colistin) [95]. Previous research has shown that carbapenem resistance in *K. aerogenes* largely stems from two main mechanisms: the upregulation of AmpC or ESBLs and mutations that alter membrane permeability. Furthermore, various carbapenemase enzymes, including NDM, KPC, and OXA-48, have also been identified in *K. aerogenes* isolates collected from patients in different countries [50,111,112]. Recently, a Chinese study conducted at a teaching hospital in Southwestern China investigated molecular epidemiology, risk factors, and clinical outcomes of 36 CRKA isolates. Their results indicated that CRKA is driven by the overexpression of AmpC and ESBLs, combined with efflux pump activity. This is the first documented case in China of a CRKA isolate simultaneously possessing *bla*_ACC_, *bla*_NDM-1_, *bla*_EBC_, *acc(6′)-Ib*, *bla*_CTX-M-15_, *armA*, and *qnrD* genes, alongside the *OmpE36* loss [113]. Interestingly, a multicenter study in the U.S. studying CREC isolates reported that the majority displayed carbapenem resistance despite not producing carbapenemases. This resistance could be linked to changes in porin channels, which are essential for antibiotic uptake, and an amplified copy number of ESBL genes [114,115].

## 8. Research Gaps

The escalating threat of AMR, particularly among ESKAPE pathogens, highlights critical research gaps. A significant challenge lies in the limited pipeline for novel antimicrobial agents, with current development failing to keep pace with rising resistance. This scarcity forces clinicians to rely on older, potentially toxic drugs with limited efficacy data. The rapid evolution of bacterial resistance mechanisms, such as target modifications, overexpression of efflux pumps, and higher enzyme production (e.g., β-lactamases), quickly renders new drugs ineffective. This scarcity is compounded by high development costs and complex regulatory hurdles, discouraging pharmaceutical investment [116]. Another major gap is the insufficient understanding of resistance mechanisms in certain ESKAPE members, such as *K. aerogenes*, where research on specific carbapenem resistance mechanisms is limited. Furthermore, the heterogeneity and diversity of resistance mechanisms within complex groups like the CREC present a significant hurdle for effective infection prevention. The global epidemiology of these pathogens, including the precise spread and evolution of specific high-risk clones and their associated resistance genes, remains largely uncertain. Another limitation is the absence of rapid and accurate diagnostic tools to identify specific ESKAPE pathogens and their resistance profiles in clinical settings, which often leads to the empirical use of broad-spectrum antibiotics, further fueling resistance. This is particularly challenging in low/middle-income countries where laboratory infrastructure is often limited [117]. Moreover, the current global surveillance systems for AMR are inadequate for the collection of comprehensive data. This limits the ability to track the emergence and spread of resistant strains, hindering timely public health interventions and policy adjustments. Alongside scientific limitations, there are several socio-economic problems like overuse and misuse of antibiotics, worldwide healthcare disparities, a general lack of public awareness and engagement, and gaps in policy-making and governance. Unawareness of public understanding about AMR, its causes, and consequences hinders the adoption of responsible antibiotic use practices and limits community engagement in AMR mitigation efforts. Despite calls for global collaboration, effective cross-border cooperation in AMR research, surveillance, and policy implementation remains a challenge, often hampered by data sharing issues, intellectual property concerns, and geopolitical complexities [118,119].

## 9. New Innovations to Combat ESKAPE Pathogens

**Figure 2:**
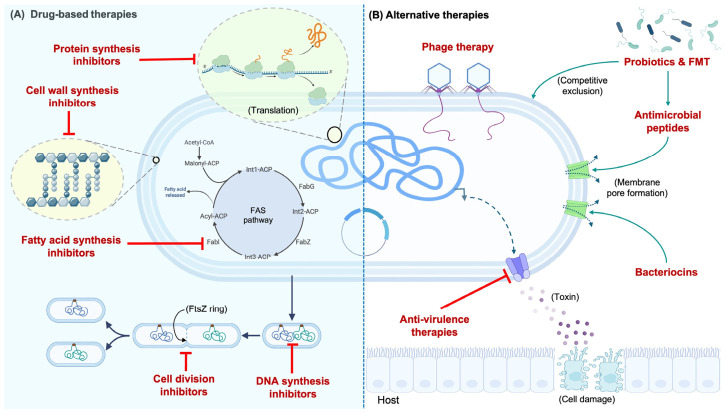
Mechanism of action of the drug-based traditional therapies and alternative non-traditional therapies against antibiotic-resistant ESKAPE pathogens. (**A**) Generally, the drug-based therapies include conventional protein synthesis inhibitors that inhibit ribosomal translation of bacterial essential proteins to prevent polypeptide chain formation; cell wall synthesis inhibitors that interfere with peptidoglycan synthesis to cause compromised cellular integrity; fatty acid synthesis (FAS) inhibitors that block the lipid biosynthesis (fatty acid) pathways; DNA synthesis inhibitors that impair the replication process; and cell division inhibitors that target proteins such as FtsZ to prevent bacterial proliferation. (**B**) On the other hand, alternative therapies include: phage therapy that specifically infects and destroys bacteria; probiotics and fecal microbiota transplantation (FMT) that competitively exclude pathogens and help restore beneficial microbiota; antimicrobial peptides (AMPs) that trigger membrane pore formation to cause cell lysis; bacteriocins that exert targeted antimicrobial effects; and anti-virulence therapies that interfere with toxin production or secretion to attenuate pathogenicity.

It is important to develop newer antibiotics and alternative therapies because of the accelerating global crisis. While antibiotics have been a foundation of modern medicine, their effectiveness is rapidly diminishing. Table 1 represents a snapshot of the ongoing efforts to develop new antibiotics, highlighting diverse mechanisms of action and targeting different resistant pathogens. Although the journey from discovery to approved drug is long and challenging, these molecules offer some hope in the fight against superbugs. The current antibiotics have a fundamental drawback of collateral damage, as traditional broad-spectrum antibiotics kill beneficial bacteria (the commensal microbiome) along with the harmful ones. This disruption compromises the natural defense system of the human body, leaving the host vulnerable to secondary infections. Therefore, there is an urgent need for the development of alternative therapies. Several innovative therapeutic strategies are currently being explored to combat AMR (Figure 2). These approaches utilize diverse mechanisms of action, ranging from direct bacterial lysis to modulating pathogen virulence and harnessing the power of the host microbiome. Some of the examples are mentioned below.

Phage therapy utilizes bacteriophages, which are viruses engineered to inject their genetic material into bacterial cells, hijacking their machinery to replicate and ultimately cause cell death. Phages exhibit high specificity to their bacterial hosts, thereby avoiding harm to commensal microbiota. Bacteriophages belonging to families such as Myoviridae, Siphoviridae, and Podoviridae exhibit specificity towards the ESKAPE pathogens. It has been reported that Myoviridae phages may infect *Staphylococcus*, *Acinetobacter*, *Pseudomonas*, and other similar bacteria. In contrast, phages belonging to the Podoviridae family primarily infect *Pseudomonas* bacteria [120,121]. Several in vitro studies have reported phages to be effective against planktonic cells and biofilm formed by the ESKAPE pathogens [122,123,124,125]. Organisms tested include *E. coli*, *S. aureus*, *P. aeruginosa*, *E. cloacae*, *K. pneumoniae*, and other MDR bacteria. Pyofag^®^ from NeoProbioCare Inc., is currently marketed for infections associated with wounds, ulcers, burns, dysbacteriosis, and acute enteric infections caused by *E. coli*, *S. aureus*, *S. pyogenes*, *P. aeruginosa*, and Proteus [126]. Clinically, several phage-based products, including Stafal by Bohemia Pharmaceuticals (Slovakia), Sextaphage Polyvalent pyobacteriophage by NPO Microgen (Russia), PhagoBioDerm by Intralytix, Inc. (U.S.), and Pyophage by the Georgian Eliava Institute of Bacteriophage, Microbiology and Virology, are commercially available and effectively combat ESKAPE pathogens. Sextaphage^®^ Polyvalent Pyobacteriophage is composed of sterile filtrate obtained from the lysate of *P. aeruginosa*, *K. pneumoniae*, *Streptococcus*, *Staphylococcus*, *Proteus vulgaris*, *P. mirabilis*, and Enteropathogenic *E. coli*. It is known to selectively inhibit *Enterococci*, *Streptococci*, *K. pneumoniae*, *Staphylococci*, *P. aeruginosa*, *Proteus*, and *E. coli* [127]. Companies such as Eliava Institute of Bacteriophages, Adaptive Phage Therapeutics (APT), Armata Pharmaceuticals (formerly AmpliPhi Biosciences), and BiomX are actively developing in this space [128,129]. A unique advantage of phage therapy is the autodosing mechanism, wherein the bacteriophage population can expand in response to the elevated bacterial load [130]. Although phages are potent interventions for treating bacterial infections, their manufacturing and formulation processes are often tedious. While phages are highly specific, their proteinogenic components (such as capsids) can occasionally trigger host immune responses, potentially compromising treatment efficacy [130]. The wide adoption of phage therapy is further complicated by significant regulatory and intellectual property hurdles, such as the difficulty in patenting naturally occurring phages and meeting EMA/FDA requirements for precise, standardized formulations. To address these limitations, the use of genetically modified phages and personalized treatment protocols is being explored to overcome the narrow host specificity inherent in wild-type varieties [131].

Antimicrobial peptides (AMPs) are naturally occurring small peptides that can modulate the host immune response, disrupt bacterial cell membranes, or interfere with essential cellular processes. They have been tested against different bacteria, including MDR strains. Many AMPs are currently undergoing preclinical to early clinical development (Phase I/II), with companies like Peptilogics and Genera Corporation, and Cutanea Life Sciences driving their research [132,133]. A proline-rich AMP dimer, A3-APO, has been shown to destabilize bacterial membranes and inhibit heat shock protein DnaK, resulting in impaired protein folding and repair processes [134]. It is reported to be efficacious against CRAB [135], MRSA [136], *K. pneumoniae*, and *E. coli* [134]. Beyond naturally derived sequences, recent advancements in protein engineering and peptide-binder engineering represent a promising frontier for designing high-efficiency targets against AMR bacteria. By utilizing these engineering pathways, researchers can move beyond wild-type limitations to create designer peptides and hybrid scaffolds—such as the K11 hybrid or fluorinated derivatives—that offer superior binding affinity and metabolic stability in the presence of ESKAPE pathogens. The K11 hybrid scaffold comprising melittin, cecropin A1, and magainin demonstrated efficacy against MDR strains of *A. baumannii* [137] and *K. pneumoniae* [138]. Recently, a novel AMP modified by the addition of fluorinated sulfono-γ-AApeptide into Feleucin-K3, CF_3_-K11, was reported to exhibit potent antibacterial activity against MRSA and *P. aeruginosa* [139]. Several other AMPs are currently undergoing development, including PT13 (plant-derived), Esc (1–21)-1c (frog skin-derived), SP-E (pig saliva-derived), SAAP-148 (inspired by LL-37 human cathelicidin), and D-150-177C, which are actively being studied for their efficacy against ESKAPE pathogens.

Anti-virulence therapies represent a different strategy by targeting bacterial virulence factors, interfering with pathogen-host interactions [140]. This approach aims to attenuate the pathogen’s ability to infect and cause damage to the host rather than directly killing it. *S. aureus* (targeting toxins, quorum sensing) [141], *A. baumannii* (targeting adhesion, iron acquisition) [142], *K. pneumoniae* (targeting capsule, adhesins) [143,144], and *C. difficile* (targeting toxins) are among the organisms tested. These therapies are largely preclinical to early clinical stages, though Bezlotoxumab (Merck & Co./Bezlotoxumab) is a clinically approved example for *C. difficile* infections [145].

Bacteriocins, which are proteinaceous toxins secreted by bacteria, can inhibit the growth of other strains by either membrane pore formation or cell wall synthesis inhibition, among other essential cellular functions. Nisin, a type of bacteriocin, exhibits wide therapeutic utility, having been advanced to Phase I clinical trials for acne treatment in conjunction with IB-367, a protegrin-like cationic peptide. Beyond dermatological applications, the Nisin variants, namely A and Z, are currently in preclinical trials focused on combating VRE. Furthermore, Nisin’s broad spectrum of activity was recently studied in a clinical trial to evaluate its pathogen-inhibitory effects associated with ventilator-associated pneumonia, caused by members of the ESKAPE [146,147,148,149].

Probiotics and fecal microbiota transplantation (FMT) both aim to restore or enhance the beneficial gut microbiota. Probiotics, which are live microorganisms, function by competitively excluding pathogens and producing AMPs (like bacteriocins) and short-chain fatty acids (SCFAs), which also serve to strengthen gut barrier functions. FMT, conversely, involves transferring fecal matter from a healthy donor to a recipient to re-establish a diverse and healthy microbial community. While recurrent *Clostridioides difficile* infections (CDI) remain the main clinical target, with VOWST and REBYOTA being examples of FDA-approved FMT products for CDI in adults following antibiotic treatment, the approach is also being investigated in Phase II/III clinical stages for other indications [150,151]. The application against multidrug-resistant ESKAPE pathogens (like CRKP and VRE) is focused on achieving gastrointestinal decolonization to prevent subsequent invasive infections. Clinical investigations, such as a prospective non-randomized comparative trial involving 48 patients, showed FMT significantly improved negative conversion rates for CRE and VRE, achieving an overall decolonization rate of 52% at 3 months compared to 24% in controls [152]. Specifically for CRKP, case reports demonstrated that a single FMT procedure could lead to complete clinical and microbiological clearance of the pathogen by restoring colonization resistance, a process supported by the ongoing randomized, double-blind, placebo-controlled KAPEDIS trial (NCT04760665) investigating encapsulated FMT for CRKP decolonization [153]. Despite ongoing investigations, having a concluding remark about the efficacy of FMT for the gastrointestinal decolonization of AMR organisms remains dubious. This limitation stems from the scarcity, low quality, and pronounced heterogeneity observed among existing reports. To accurately guide clinical practice, further research must prioritize rigorous, large-sample randomized controlled trials with strictly standardized protocols [152].

CRISPR-based therapies against ESKAPE pathogens are predominantly in the preclinical and early clinical trial stages, focusing on two main strategies: genome editing for re-sensitization and targeted cell killing [154,155,156]. The most advanced approach is the use of the CRISPR-Cas system to specifically eliminate the resistance genes found on mobile genetic elements (plasmids), a process known as plasmid curing or gene-focused cleavage. For instance, a proof-of-concept study demonstrated that a novel CRISPR-Cas9-mediated plasmid-curing system (pCasCure) could effectively cure the *bla*_NDM_, *bla*_KPC_, and *bla*_OXA-48_ from clinical isolates of CRKP and other Enterobacteriaceae, restoring their susceptibility to carbapenems [157,158]. Furthermore, other approaches involve using CRISPR-dCas9 (a deactivated nuclease) to suppress the transcription of resistance genes, as was studied against the *mecA* gene in MRSA to reduce antimicrobial resistance without immediately killing the bacteria [159]. Finally, Locus Biosciences conducted a Phase 1b trial (NCT04746973 for *E. coli*, closely related to *Enterobacter* spp.) using a bacteriophage-delivered CRISPR-Cas3 system to treat urinary tract infections (UTIs), a common site for ESKAPE pathogens, which supported the safety and tolerability of this first CRISPR-based therapy for infection.

## 10. Path to a Better Future

Addressing antimicrobial resistance represents a formidable global challenge necessitating a concerted, multi-sectoral approach involving individuals, healthcare systems, national governments, and international organizations. The foundational strategy guiding these efforts is commonly termed “One Health”, which underscores the inextricable interconnectedness of human, animal, and environmental health. Effective mitigation of AMR is being pursued through several critical strategic pillars: enhancing public and professional awareness and comprehension of the issue; bolstering the foundational knowledge and evidence base through rigorous research and data collection; proactively reducing the incidence of infections to diminish the reliance on antimicrobials; optimizing the judicious use of existing antimicrobial agents to prevent their misuse; and cultivating a robust economic framework to secure sustained investment in novel treatments and intervention strategies (details are given in Table 2). Beyond global policy, localized clinical strategies such as antibiotic short- and long-term cycling (or rotation) are sometimes implemented within hospital settings. These protocols involve periodically alternating the primary classes of antibiotics used in specific wards to disrupt the persistent selective pressure on local microbial populations and prevent the emergence of nosocomial resistance. Through the concentrated application of these interdependent approaches, the global community endeavors to decelerate the emergence and spread of resistant pathogens.

## 11. Discussion

The global rise of AMR constitutes a critical public health crisis, dramatically weakening the effectiveness of current drugs and escalating the risk of disease transmission and infection-related deaths [1]. This pervasive challenge is mainly driven by the misuse and overuse of antimicrobials, combined with poor adherence to treatment protocols and inadequate diagnostic tools. AMR also creates a significant economic burden through extended hospital stays, high-cost medicines, and complex treatment regimens. Crucially, the rate of resistance development (e.g., by increasing efflux pump expression and higher production of β-lactamase enzymes) has surpassed the new drug development pipeline, leading to a substantial decrease in the antibiotic development pipeline [6]. In response to this urgent threat, the WHO has prioritized a group of pathogens known as ESKAPE, listed in its 2017 and 2024 bacterial priority pathogens reports [5,7]. The ESKAPE are responsible for a significant proportion of nosocomial infections and present major therapeutic challenges in clinical settings due to their extensive resistance profiles [116]. ESKAPE pathogens include both Gram-positive and Gram-negative organisms with distinct mechanisms of drug resistance.

Despite vancomycin being a last-resort treatment for severe Gram-positive infections, VRE prevalence has become high in certain global regions, demonstrating an alarming increase in prevalence over the past two decades in countries like India. This resistance is mediated by genes like *vanA* or *vanB*, where the VanA ligase catalyzes the substitution of the peptidoglycan precursor D-Ala-D-Ala terminus with D-Ala-D-Lac, which prevents vancomycin from binding to the cell wall [17,160]. Similarly, *S. aureus* causes severe infections, often complicated by its ability to form biofilms. MRSA resistance is conferred by the *mecA* and *mecC* genes, which encode the PBP2a and PBP2c, providing resistance against β-lactam antibiotics. Subsequent widespread vancomycin use against MRSA has led to the emergence of strains with reduced susceptibility: VISA and VRSA [56]. The frequency of both VISA and VRSA has increased substantially globally [52]. VRSA exhibits full resistance, while VISA demonstrates intermediate resistance. This resistance is typically mediated by the acquisition of the *vanA* operon from the Enterococcus reservoir via the transposon Tn1546 [60,62,63].

Carbapenem resistance poses a major clinical challenge across several Gram-negative ESKAPE pathogens, which frequently cause HAIs in vulnerable patients [68]. CRAB, an opportunistic coccobacillus, exhibits resistance primarily through the high production of carbapenemases (like *OXA*-type enzymes such as *bla*_OXA-23_ and *bla*_OXA-24/40_) and MBLs (e.g., IMP, VIM, and NDM). This resistance is compounded by changes like the modification of PBPs, overexpression of efflux pumps, and reduced permeability via OMP defects (e.g., *CarO*). Most CRAB isolates worldwide belong to a few dominant International Clones (ICs), with one clone (IC2) being the most prevalent globally [71,74]. Similarly, CRPA, which infects immunocompromised hosts, displays a high and increasing prevalence in many regions. CRPA utilizes multiple resistance strategies, including increased activity of the MexA-MexB-OprM efflux pump, elevated *AmpC* production, and the inactivation of the *OprD* porin, with MBL production (like VIM) often being the primary driver of resistance spread via mobile genetic elements [89,90]. Finally, CRE, which includes CRKP [101] and *E. cloacae* (CREC), is showing very high rates of resistance in several countries. The key mechanism here is the production of β-lactamase enzymes, encompassing ESBLs and various carbapenemases (serine types like KPC and OXA-48-like, and MBLs like NDM, VIM, and IMP). However, resistance can also arise in some CRE isolates through porin channel changes and amplified ESBL gene copy numbers even without carbapenemase production [109,110,111].

The urgent need for alternative therapeutic strategies against AMR is driven by the high cost and low return on investment associated with traditional antibiotic development, necessitating approaches that can overcome the limitations of existing drugs [128]. One such strategy is phage therapy, which employs specific bacteriophages (viruses from families such as *Myoviridae*, *Siphoviridae*, and *Podoviridae*) to target and lyse ESKAPE pathogens, including *S. aureus*, *Acinetobacter,* and *P. aeruginosa*; this approach benefits from an autodosing mechanism where phages replicate in response to the bacterial load, with commercial products available for pathogens like *P. aeruginosa* and *K. pneumoniae*. Another promising avenue involves antimicrobial peptides (AMPs), small naturally occurring peptides that disrupt bacterial membranes; examples include the proline-rich AMP dimer A3-APO and the synthetic K11 hybrid scaffold, both of which have demonstrated efficacy against multidrug-resistant ESKAPE members such as CRAB, MRSA, and CRKP. Additionally, FMT, while primarily used for recurrent *C. difficile* infections, is being investigated for the treatment of MDR-ESKAPE pathogens like CRKP and VRE to prevent invasive infections, showing improved negative conversion rates for CRE and VRE colonization in comparative trials. Finally, CRISPR-based therapies represent an advanced approach that leverages genome editing for re-sensitization or targeted cell killing, with studies demonstrating that plasmid curing systems, CRISPR-Cas9, can effectively eliminate carbapenemase genes (*bla*_NDM_, *bla*_KPC_, and *bla*_OXA-48_) from clinical isolates of CRKP and other Enterobacteriaceae, successfully restoring antibiotic susceptibility.

Ultimately, combating the multifaceted threat of AMR among ESKAPE pathogens necessitates a holistic, multi-sectoral approach guided by the One Health principle, which dictates that strategies must address the interconnectedness of environmental, human, and animal health by optimizing antibiotic usage, strengthening surveillance and research, and ensuring sustainable investment in new interventions.

## 12. Conclusions

We believe this article provides a valuable and comprehensive examination of the critical global threat posed by AMR, specifically focusing on the WHO priority list of ESKAPE pathogens. The strength and uniqueness of our analysis lie in our in-depth dual approach, as we first thoroughly detail the current global prevalence, specific antibiotic-resistant patterns, and underlying molecular mechanisms of both Gram-positive (VRE, MRSA, and VISA/VRSA) and Gram-negative Carbapenem-Resistant (CRAB, CRPA, and CRE/CRKP) ESKAPE organisms. This comprehensive mechanistic review directly addresses the challenge arising from the decline in the traditional antibiotic development pipeline. Furthermore, our unique contribution is our extensive discussion and compilation of innovative non-traditional therapies being explored to overcome resistance. These cutting-edge alternatives include phage therapy, antimicrobial peptides (AMPs), anti-virulence therapies, fecal microbiota transplantation (FMT), and targeted CRISPR-based approaches, offering a forward-looking perspective on mitigating this pervasive crisis.

## Figures and Tables

**Table 1:** Novel drugs with the mechanism of action and current clinical phase.

Class	Drug Molecule	Details	Organisms Against	Clinical Phase	Company/ Developer
Cell wall synthesis inhibitor	Zosurabalpin	Disrupts outer membrane lipoprotein transport (LptB2FGC complex), preventing LPS assembly in Gram-negative bacteria	CRAB	Phase III	Roche
	Cefepime-Taniborbactam	Cefepime is β-lactam combined with Taniborbactam a, broad-spectrum β-lactamase inhibitor	ESBL, CRE, MDR *Pseudomonas*, MBL-producing pathogens, cUTIs	Phase III (NDA submitted)	Venatorx Pharmaceuticals/Everest Medicines
	Nacubactam (OP0595)	Inhibits serine and metallo-β-lactamases	Carbapenem-resistant Enterobacterales, *P. aeruginosa*, *A. baumannii*	Phase II	Fedora Pharmaceuticals; Meiji Seika Pharma
	Sulbactam-Durlobactam (Xacduro)	Sulbactam (β-lactam) combined with Durlobactam (novel β-lactamase inhibitor)	CRAB	Approved (FDA 2023)	Entasis Therapeutics
	Fosfomycin IV (repurposed)	Binds to the MurA enzyme and inhibits early steps of cell wall synthesis	Multi-drug-resistant Gram-negative bacteria	Phase III (new formulations/indications)	Pfizer, and Spero
	Taniborbactam	Broad-spectrum β-lactamase inhibitor (including activity against MBLs)	Gram-negative pathogens	Phase III (in combinations)	Venatorx Pharmaceuticals
	Darobactin	Inhibits BamA and disrupts the proper formation of the Gram-negative cell envelope	*P. aeruginosa*, *A. baumannii* Enterobacterales	Early Clinical	DZIF/HIPS (German Research Centre)
	VNRX-7145 (oral)	Oral prodrug of a novel β-lactamase inhibitor	ESBL-producing Enterobacterales, cUTI	Phase I/II	Venatorx Pharmaceuticals
DNA Synthesis inhibitor	SPR720	Inhibits DNA gyrase and topoisomerase IV	Non-tuberculous mycobacteria (NTM), e.g., *M. avium*	Phase II	Spero Therapeutics
	Gepotidacin	Inhibits DNA gyrase and topoisomerase IV	*Neisseria gonorrhoeae*	Phase III	GSK
	Delafloxacin	Inhibits DNA gyrase and topoisomerase IV	ABSSSI, CABP MRSA	Approved for ABSSSIs and is being studied for community-acquired pneumonia.	Melinta Therapeutics
Protein synthesis inhibitor	NXL104	Binds to 23S rRNA (macrolid)	CAP, multidrug-resistant *S. pneumoniae*	Phase III	Cempra/Nabriva Therapeutics
	Cresomycin	A bridged macrobicyclic antibiotic that can bind to the bacterial ribosome	Drug-resistant *S. aureus*, *P. aeruginosa*, other Gram-negative and Gram-positive bacteria	Early Clinical	Harvard University/CARB-X funded programs
Outer Membrane Modulator	Murepavadin	Antimicrobial peptidomimetic which specifically targets the LptD protein in outer membrane of *P. aeruginosa* and blocks LPS transport	MDR *Pseudomonas aeruginosa*	Phase II	Spexis (formerly known as Polyphor)
Fatty acid synthesis inhibitor	AFN-1254	Binds to Enoyl-acyl carrier protein reductase enzyme (FabI)	MRSA and VRSA	Phase II	Affinium Pharmaceuticals

Bacterial skin and skin structure infections (ABSSSI), community-acquired bacterial pneumonia (CABP), and community-acquired bacterial pneumonia (CAP).

**Table 2:** Socio-economic measures to combat AMR globally.

Pillar	Key Strategy	Description
Awareness	Public Awareness Campaigns	Educating people about the dangers of AMR.
	Educating Healthcare Professionals	Training doctors, nurses, pharmacists, and veterinarians on responsible antimicrobial prescribing practices.
Strong Knowledge Base	Surveillance & Research	Establishing national and international surveillance systems to monitor antibiotic consumption patterns and track the emergence and spread of resistant microbes.
	Rapid Diagnostics	Developing and implementing faster and more accurate diagnostic tests to identify infections and determine appropriate treatments.
Reduced Infection	Infection Prevention & Control	Reinforcing strict hygiene practices in healthcare settings.
	Vaccination	Promoting and ensuring easy access to existing vaccines, thereby preventing infections and reducing antibiotic usage.
	Sanitation & Hygiene	Investing in better sanitation, clean water, and food safety practices.
	Biosecurity in Agriculture	Implementing measures to prevent and control infections in animals.
Optimized Antibiotic Usage	Appropriate Prescribing	Ensuring that antibiotics are only prescribed when necessary, for the correct duration, and at the appropriate dose. Implementing local antibiotic rotation/cycling protocols.
	Limiting Over-The-Counter (OTC) Sales	Restricting access to antibiotics without a prescription to prevent misuse and overuse.
	Developing Guidelines	Creation of strict clinical guidelines for antimicrobial use in human and animal health.
	Reducing Agricultural Usage	Discontinue the use of antibiotics for growth promotion in healthy animals.
Sustainable Investment	Research & Development	Increasing investment in the discovery and development of new antibiotics, diagnostic tools, vaccines, and alternative therapies.
	Sustainable Financing	Support AMR initiatives in low- and middle-income countries.
	Global Collaboration	Fostering intergenerational partnerships and a global coalition for action, as AMR is a global threat.
	National Action Plans	Supporting countries to develop and implement a comprehensive national action plan on AMR aligned with the Global Action Plan.

## Data Availability

No new data were created or analyzed in this study. Data sharing is not applicable to this article.
